# Donor–Recipient Race Mismatch Is Associated with Lower Survival After Liver Transplantation for Primary Sclerosing Cholangitis

**DOI:** 10.3390/jcm14155441

**Published:** 2025-08-01

**Authors:** Mark W. Russo, Will Wheless, Wida S. Cherikh, Alice E. Toll, Alexandra T. Lewis, Andrew S. deLemos

**Affiliations:** 1 Division of Hepatology, Department of Medicine, Atrium Health Wake Forest, Charlotte, NC 28204, USA; william.wheless@atriumhealth.org (W.W.); andrew.delemos@atriumhealth.org (A.S.d.); 2 Research Department, United Network for Organ Sharing, Richmond, VA 23219, USA; wida.cherikh@unos.org (W.S.C.); alice.toll@unos.org (A.E.T.); alexandra.lewis@unos.org (A.T.L.)

**Keywords:** outcomes, graft, patient, survival, outcomes, race, ethnicity

## Abstract

**Background:** Patient survival after liver transplantation is lower in donor–recipient race mismatched patients for indications other than primary sclerosing cholangitis. **Objectives**: To determine if survival is lower after liver transplantation in donor–recipient race mismatched recipients with primary sclerosing cholangitis. **Methods**: The Organ Procurement and Transplantation Network database was analyzed for deceased donor adult liver transplant recipients with primary sclerosing cholangitis. Graft and patient survival by donor–recipient race were estimated using Kaplan–Meier survival method and compared using the log-rank test. Multivariable analysis was performed using Cox regression. **Results**: From 2002 to 2018, 5-year patient survival in White (*n* = 2223) and Black recipients (*n* = 491), was 89.8% and 87.1%, respectively. Five-year patient survival for the donor–recipient pairs, White–White (*n* = 1622), Black–Black (*n* = 110), Black–White (*n* = 335), and White–Black (*n* = 314) was 90.8%, 91.1%, 87.1%, and 86.0%, respectively, *p* = 0.026. In multivariable analysis, 5-year patient mortality was higher in Black recipients of White donors [HR 1.69, 95% CI 1.16, 2.45], compared to White recipients of White donors. **Conclusions**: Five-year patient mortality after deceased donor liver transplantation for primary sclerosing cholangitis is higher in Black recipients who received livers from White donors compared to matched White donors and recipients.

## 1. Introduction

In 2023 there were 371 adult patients with end-stage liver disease from primary sclerosing cholangitis (PSC) listed for liver transplantation and 385 adult liver transplants performed for PSC in the United States [1]. PSC is an indication for liver transplantation worldwide. The crude incidence of liver transplantation in the United Kingdom was 10.9 per 1000-person-years while in the Netherlands 94 of 590 transplants were performed for end-stage liver disease from PSC over a median follow-up of 92 months [2]. Liver transplantation for PSC is associated with excellent 1-year patient and graft survival exceeding 95%; however, recurrent PSC occurs in up to 20% of recipients within five years of transplant [3]. Studies of liver transplantation and autoimmune or cholestatic liver diseases typically combine results of PSC, primary biliary cholangitis and autoimmune hepatitis or do not include diverse patient populations [3,4,5,6,7].

The development of effective medical therapy for PSC before or after liver transplantation has been challenging. Effective therapy is needed because recurrent PSC may progress to graft failure after liver transplantation. Factors associated with graft failure or recurrent disease after liver transplantation include recipient age, donor age, rejection episodes, donor after circulatory death, ulcerative colitis, and history of colectomy prior to transplants [4,5,6,7]. Adult liver recipients of living and deceased donor liver transplants with PSC have a similar risk of recurrent disease after liver transplantation [8]. Donor–recipient sex mismatch has not been associated with recurrent PSC or survival after liver transplantation for PSC [9].

Donor–recipient race mismatch has been associated with outcomes after liver transplant [10,11,12]. An analysis of the Organ Procurement and Transplantation Network (OPTN) database reported that race matched Caucasian recipients experienced a 1–3% improvement in mortality in lung, liver, and pancreas transplants [10]. Among recipients transplanted for a variety of indications, matched Black donor–recipients experienced a 4–6% improvement in patient and graft survival [10]. Black recipients of White donors have a 1.39-fold increase in graft loss compared to donor–recipient matched liver transplants [13]. These studies have not reported outcomes in patients transplanted for end-stage liver disease from PSC. Because studies have demonstrated differences in survival after liver transplantation in donor-race mismatched recipients for indications other than PSC, we sought to report outcomes after liver transplantation for by donor and recipient race.

## 2. Methods

We analyzed the OPTN database for adult (≥18 years old) recipients of deceased donor liver transplants with diagnosis of end-stage liver disease and cirrhosis from PSC between 27 February 2002 (implementation of Model for End-Stage Liver Disease (MELD) and 26 February 2020 (prior to COVID pandemic) who were alive with a functioning graft at discharge. Recipients that had a diagnosis of PSC with or without inflammatory bowel disease were included. Multiorgan transplants, retransplants, and individuals with a prior transplant were excluded. Two transplant eras (2002–2011 and 2012–2020) were used to evaluate changes in characteristics of all PSC transplants over time while still maintaining an adequate number of recipients in each era, including for subgroup analyses. Our study complied with the strengthening the reporting of observational studies in epidemiology (STROBE) guidelines [14] (Supplementary Table S1).

The primary outcomes were defined as graft failure and death within 5 years after transplantation, by donor–recipient race. Follow-up time was measured as the duration from the transplant date to either the occurrence of the primary outcomes or the last known follow-up date. For 5-year graft and patient survival outcomes were assessed for adult deceased donor liver transplants through September 2018, to allow for 5 years of follow-up data plus an additional 3 months due to data reporting lags. We only considered graft failure and death within 5 years of transplant; any transplant recipient who had not experienced either event by the end of the five-year period was administratively censored.

Primary outcomes of graft failure and death within 5 years of transplant were analyzed from 2012 to 2018 as well as stratified by era (era 1: 2002–2011 vs. era 2: 2012–2018). Secondary outcomes included 1-year graft and patient survival by recipient race and ethnicity were analyzed from 2012 to 2020. Race and ethnicity were self-identified. Donor and recipient race was classified as White, Black, Hispanic, Asian, and ‘Other” in the univariate tabulation. For the outcomes analysis, the Hispanic and Asian categories were combined into ‘Other’ due to sample size. Additionally, donor-to-recipient race was categorized as White–White, White–Black, Black–White, and ‘Other’, with ‘Other’ encompassing any combinations outside the first four. A donor–recipient mismatch refers to transplant where the race of the donor differs from that of the recipient. The use antibody induction drug at transplant was categorized as IL (interleukin)-2 receptor antibody (basiliximab, daclizumab), monoclonal (alemtuzumab), polyclonal (anti-thymocyte globulin), or none. Maintenance immunosuppressive drugs were summarized as the usage of tacrolimus and mycophenolate mofetil at discharge.

The current analysis was performed using OPTN data as of 5 April 2024. The data were analyzed using R version 4.3.3, R Project, Vienna, Austria.

## 3. Statistical Methods

Continuous variables were summarized as median and interquartile range (IQR). Distributions of continuous variables were compared by group using a Kruskal–Wallis test. Categorical variables were reported as number and percent, and the distribution was compared using a Chi-square test. Unadjusted Kaplan–Meier models were used to estimate patient and graft survival within 5 years and compared by transplant group using log-rank tests. *p*-values were adjusted for multiple comparisons of survival using the Benjamini–Hochberg procedure. Risk adjustment variables used in the Cox models were identified a priori to reduce bias.

The variables were selected based on clinical knowledge and the results of the univariate analyses. The primary covariate of interest in the Cox model was donor–recipient race mismatch, and the additional risk adjustment variables were transplant era, recipient age, donor age, liver donor risk index (LDRI), and treatment for acute rejection at discharge. Five-year patient and graft survival are reported for adult deceased donor liver transplants from 2002 to 2018. Our multivariable cohort had 10.9% missingness for these covariates of interest. Missing values for covariates were imputed using multiple imputation by chained equations via the aregImpute function from the Hmisc package version 5.1–3 and pooled across 11 imputation datasets for the cohort of deceased donor transplants. The results from each imputed dataset were combined to produce final estimates, including hazard ratios and *p*-values. Cox models for patient and graft survival were fit among deceased donor transplants, with administrative censoring at 5 years applied. The model with donor–recipient race mismatch utilized 14 degrees of freedom.

## 4. Results

During 2002–2020, 3790 adults underwent liver transplantation for end-stage liver disease from PSC. Characteristics of recipients included a median age of 48 years old, 31.6% female, 15.2% Black, 4.2% Hispanic or Latino, 84.4% deceased donor recipients, 15.6% adult living donor recipients, 4.4% donor after circulatory death recipients, and median MELD allocation score of 23 (Table 1). Tacrolimus and mycophenolate mofetil were the most common discharge maintenance immunosuppressants, in 3641 (96.1%) and 3053 (80.6%) recipients, respectively. Overall 1-year graft and patient survival were 94.6% [95% CI 93.8–95.4%] and 97.2% [95% CI 96.6–97.8%], respectively.

Five-year patient and graft survival was available for 2907 adult deceased donor recipients who underwent liver transplant from 2002 to 2018. Overall 5-year graft and patient survival were 83.2%, [95% CI 81.9–84.6%] and 89.0% [95%CI 87.9–90.2%], respectively. The most common identified causes of death within 5 years of transplant in descending order were malignancy, infection, graft failure, and multiorgan system failure. Recurrent disease as a cause of graft failure within 5 years of transplant was reported in 13.7% of cases.

**Figure 1:**
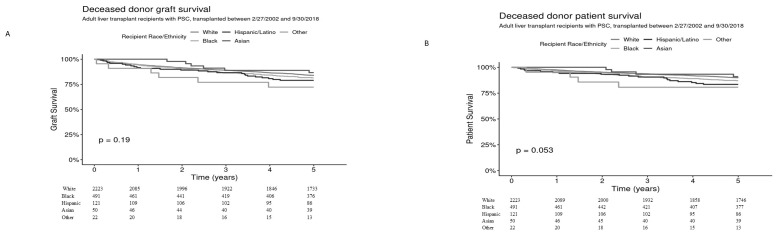
Graft (**A**) and patient (**B**) survival for adult liver transplants from deceased donors for recipients with PSC, by race and ethnicity.

Graft and patient survival within 5 years was assessed by recipient race and ethnicity among 2907 deceased donor transplants. Five-year graft survival for White (*n* = 2223), Black (*n* = 491), Hispanic/Latino (*n* = 121), Asian (*n* = 50), and Other (*n* = 22) was 83.9% [95% CI 82.3–85.4%], 81.5% [95% CI 78.1–85.0%], 79.0% [95% CI 72.0–86.7%], 86.7% [95% CI 77.3–97.2%], and 72.2% [95% CI 55.5–93.9%], respectively (overall *p* = 0.19) (Figure 1A) and 5 year-patient survival was 89.8% [95% CI 88.5–91.1%], 87.1% [95% CI 84.1–90.1%], 83.5% [95% CI 76.9–90.6%], 90.9% [95% CI 82.8–99.8%], and 80.7% [95% CI 65.3–99.6%], respectively, *p* = 0.053 (Figure 1B).

### 4.1. Donor and Recipient Race

For deceased donor transplants donor–recipient race was White–White (W-W) (*n* = 1622), Black–Black (B-B) (*n* = 110), Black–White (B-W) (*n* = 335), and White–Black (W-B) (*n* = 314). Donor age was youngest in Black donors to Black recipients and allocation MELD score was highest in White donors to Black recipients (Table 2).

**Figure 2:**
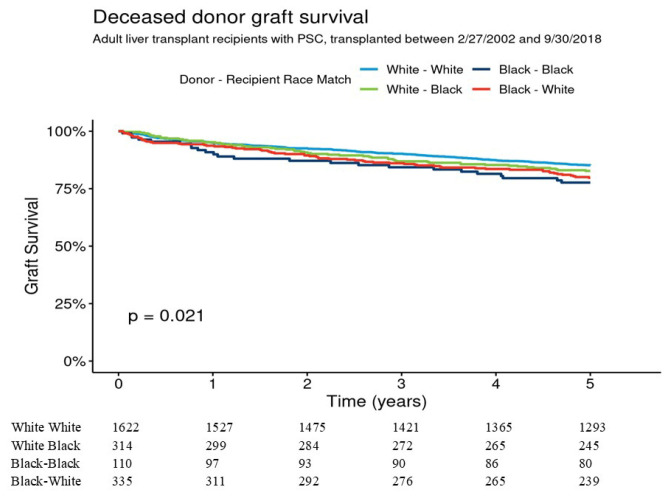
Graft survival for adult liver transplants from deceased donors for recipients with PSC, by donor–recipient race.

**Figure 3:**
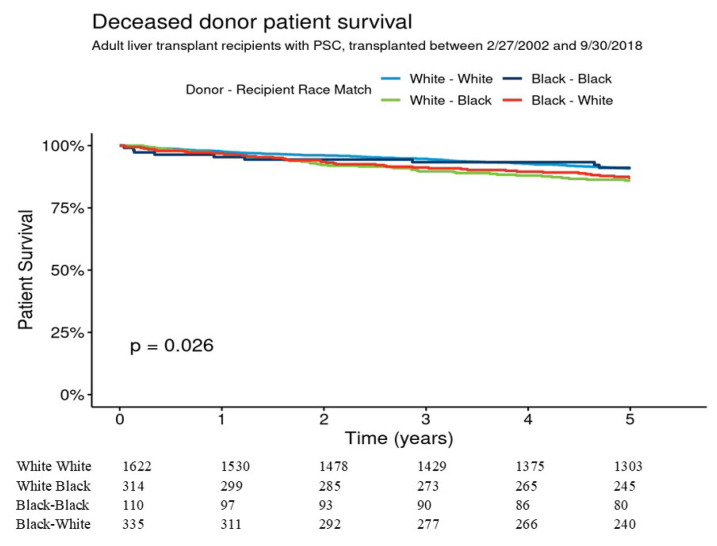
Patient survival for adult liver transplants from deceased donors for recipients with PSC, by donor–recipient race.

Five-year graft survival for W-W, B-B, B-W, and W-B was 85.2% [95% CI 83.4–86.9%], 77.7% [95% CI 70.2–85.9%], 79.7% [95% CI 75.4–84.2%], and 82.7% [78.6–87.0%], respectively (overall *p* = 0.021) (Figure 2). Pairwise comparisons indicated that the graft survival differences were not significant for W-W vs. B-B (*p* = 0.09) and for W-W vs. B-W (*p* = 0.08). In multivariable analysis 5-year graft survival was not significantly different between B-B, B-W, and W-B as compared to W-W donor–recipient group. Five-year patient survival for W-W, B-B, B-W, and W-B were 90.8% [95% CI 89.3–92.2%], 91.1% [95% CI 85.7–96.7%], 87.1% [95% CI 83.5–90.9%], and 86.0% [95% CI 82.2–89.9%], respectively, *p* = 0.026 (Figure 3). Pairwise comparisons showed that patient survival difference was marginally significant for W-W vs. W-B donor–recipient group (*p* = 0.051). The adjusted 5-year risk of patient mortality was 1.69-fold higher in the W-B donor–recipient group compared to the W-W donor–recipient group (HR = 1.69 [95% CI 1.16–2.45], (*p* = 0.006) (Table 3).

### 4.2. Era 1 Vs. Era 2

The number of transplants from 2002 to 2011 (era 1) and 2012 to 2020 (era 2) were 1890 (49.9%) and 1900 (50.1%), respectively. From year to year the number of patients transplanted for PSC was similar ranging from 168 to 285 recipients or 3.6–5.6% of total liver transplants. Median recipient age was similar between the two eras while the proportion of female recipients increased from 29.8% to 33.4% (*p* = 0.02). Allocation MELD score increased from era 1 to era 2, 21 and 25, respectively (*p* < 0.001). More recipients received induction therapy and MMF discharge maintenance for immunosuppression in era 2 than era 1, 89.1% and 72.0%, respectively (*p* < 0.001) (Table 1). Overall graft and patient survival rates within 5 years of transplant among deceased donor transplants between 2002 and 2018 were not significantly different between era 1, 88.6% [95% CI 87.0–90.2%] and era 2, 89.6% [95% CI 87.9–91.3%], *p* = 0.31.

The proportion of White patients decreased from era 1 to era 2, 1536 (81.3%) to 1423 (74.9%), respectively, while the proportion of Black, Asian, and Hispanic/Latino patients transplanted for PSC increased, 255 (13.5%) to 321 (16.9%), 26 (1.4%) to 42 (2.2%), and 64 (3.4%) to 97 (5.1%), respectively (*p* < 0.001).

## 5. Discussion

Liver transplantation for end-stage liver disease from primary sclerosing cholangitis is associated with excellent outcomes at 1-year, but recurrent PSC occurs in 15–20% of recipients within 5 years of transplantation. Studies have reported factors associated with recurrent PSC, however they have not reported outcomes by race or ethnicity or by donor–recipient race which has impacted transplant outcomes for other diseases [3,4,5,6,7,8]. Our study was conducted across two decades that provides outcomes five years after liver transplant in a diverse patient population. While the scientific registry for transplant recipients report provides national data for liver transplantation, it combines PSC with other autoimmune or cholestatic diseases including autoimmune hepatitis and primary biliary cholangitis and does not report outcomes by donor and recipient race.

The higher patient mortality in donor–recipient race mismatched patients with PSC is a novel finding. Five-year patient mortality for deceased donor transplants was 1.69-fold higher in Black recipients from White donors compared to White recipients from White donors. The higher mortality is not explained by older donor age, which was adjusted for in multivariable analysis. Others have reported worse outcomes in race mismatched liver transplants, including hepatitis C where one explanation was differences in expression of the IL-28B polymorphism. Human leukocyte antigen mismatching has not been associated with rejection or graft survival after liver transplantation for autoimmune liver diseases [15,16]. Our study did not find a significant difference in 5-year graft survival in donor-race mismatched recipients. The discrepancy between a difference in patient survival but not graft survival may be due to unrecognized confounding factors. Due to limitations in data available in the organ procurement and transplantation network database (OPTN) there may be other variables not accounted for, such as nutritional status or patient compliance with immunosuppression and follow-up, which explain the association between higher patient mortality in Black recipients of White donors. Nevertheless, our findings warrant further study to uncover the reasons for higher mortality in Black recipients from White donors.

Our study demonstrates the changing racial and ethnic makeup of patients transplanted for PSC in the United States. The relative proportion of White patients transplanted for PSC decreased from era 1 to era 2 by 7.9%, while the relative proportion of Black, Asian, and Hispanic or Latino patients increased by 25.2%, 57.1%, and 50%, respectively. These changes are not completely explained by changes in demographics of the U.S. population. From 2010 to 2020 there was a 5.6%, 35.5%, and 23% increase in the Black, Asian, and Hispanic or Latino populations, respectively [17]. A possible explanation for the increase in liver transplantation for PSC in non-White patients is the implementation of the Affordable Care Act (ACA) and Medicaid expansion that occurred during and after 2010 which may have resulted in increased access to care. The greatest reductions in uninsured rates occurred in Black, Asian and Hispanic populations. From 2010 to 2022 the percent of uninsured White, Black, Asian, and Hispanic individuals decreased from 13.1% to 6.6%, 19.9% to 10.0%, 16.7% to 6.0%, and 32.6% to 18.0%, respectively [18]. It is possible that the increase in liver transplants in patients with PSC is partly attributed to increased access to health care and liver transplantation after introduction of the ACA. While this may be an encouraging finding from our work, addressing the structural racism that exists in access to liver transplant remains critically important [19]. We can only speculate on other explanations for the increase in liver transplants for PSC in Black, Asian and Latino populations including better awareness of autoimmune liver diseases, an increase in access and referrals to gastroenterologists and hepatologists, or an increase in the number of hepatologists in the United States

Factors other than donor–recipient race that are associated with recurrent PSC or graft survival have been reported. In a meta-analysis that included 14 studies and 2481 recipients transplanted for PSC variables associated with recurrent disease included colectomy prior to liver transplant, inflammatory bowel disease, cholangiocarcinoma, donor age, acute cellular rejection, and MELD score [20]. Colectomy prior to transplant was associated with a 35% reduced risk of recurrent PSC while acute cellular rejection, cholangiocarcinoma and inflammatory bowel disease were associated with an increased risk of recurrent PSC. The meta-analysis included studies from the U.S. as well as other countries where allocation systems may be different than the U.S. Additionally, the inclusion period for most of the studies started in the 1980s when tacrolimus may not have been widely available and cyclosporine use was more common which may have impacted the rate of rejection or graft loss. Studies included in the meta-analysis did not evaluate the association between survival and donor–recipient race.

Limitations of our study include data availability in the OPTN database. Although our primary outcome was graft and patient survival, we could not reliably determine the cause of graft loss or patient death. The study was limited to five years and results may not capture grafts that would be potentially lost to recurrent PSC or how many recipients died from recurrent PSC or related conditions as well as those who may be too unwell to be re-transplanted. The OPTN database provides a large, population-based cohort for liver transplant recipients, but it does not provide granular detail for many variables including for precise causes of death or data may be missing or inaccurate. However, data for variables we were able to include in our model were missing in 10% or fewer cases. There were too few cases to stratify donor–recipient race in living donor liver transplant recipients with PSC and limited data on ethnicity so our results may not be generalizable to these populations. We did not validate our findings in an independent cohort of recipients and our findings of donor–recipient race mismatch on patient survival warrants validation prior to reaching definitive conclusions. However, the cohort included a large population of Black patients with PSC which has been a limitation of other studies.

In conclusion, our study provides useful data across two decades on outcomes for adults undergoing liver transplantation for PSC, by donor and recipient race. The results demonstrate an association between donor–recipient race mismatch and patient survival which warrants further study. In addition, over time more Black individuals have undergone liver transplantation for end-stage liver disease from PSC in the United States. With changing demographics and the challenges associated with managing recurrent PSC after liver transplant, it is important to pursue further research to validate our findings and investigate possible causes of lower patient survival in donor–recipient race mismatched liver transplant recipients.

## Figures and Tables

**Table 1:** Characteristics of the study population among adult liver recipients with PSC transplanted during February 2002–February 2020, by transplant era.

	Total *n* = 3790	Era 1 (2002–2011) *n* = 1890	Era 2 (2012–2020) *n* = 1900	*p*-Value
Age (median, IQR)	48 (36, 58)	49 (37, 58)	48 (36, 59)	0.897
Female (%)	1197 (31.6)	782 (41.4)	829 (43.6)	0.02
White (%)	2959 (78.1)	1536 (81.3)	1423 (74.9)	<0.001
Black (%)	576 (15.2)	255 (13.5)	321 (16.9)	
Asian (%)	68 (1.8)	26 (1.4)	42 (2.2)	
Hispanic (%)	161 (4.2)	64 (3.4)	97 (5.1)	
Other (%)	26 (0.6)	9 (0.5)	17 (0.9)	
BMI (IQR)	24.6 (22.1, 27.9)	24.6 (22.1, 27.8)	24.6 (22.1, 28.0)	
Allocation MELD (IQR)	23 (18,29)	21 (17, 26)	25 (20, 32),	<0.001
Donor type				0.135
Deceased donor (%)	3198 (84.4)	1612 (85.3)	1586 (83.5)	
Living donor (%)	592 (15.6)	278 (14.7)	314 (16.5)	
DCD (%)	142 (4.4)	65 (4.0)	77 (4.9)	0.297
Diabetes (%)	391 (10.4)	186 (10.0)	205 (10.8)	0.449
Ascites (%)	2436 (64.6)	1271 (67.9)	1165 (61.3)	<0.001
Encephalopathy (%)	1731 (45.9)	885 (47.2)	846 (44.5)	0.106
Induction type				<0.001 <0.001
IL-2	549 (14.5)	229 (12.1)	320 (16.8)	
Monoclonal	53 (1.4)	44 (2.3)	9 (0.5)	
Polyclonal	340 (9.0)	129 (6.8)	211 (11.1)	
None	2848 (75.1)	1488 (78.7)	1360 (71.6)	
Tacrolimus (%)	3641 (96.1)	1786 (94.5)	1855 (97.6)	<0.001
MMF (%)	3052 (3.9)	1360 (72)	1693 (89.1)	<0.001
Donor age (median, IQR)	40 (26, 53)	40 (25,53)	40 (27, 53)	0.586
LDRI (median, IQR)	1.35 (1.11, 1.63)	1.33 (1.10, 1.63)	1.35 (1.11, 1.63)	0.134
Cholangiocarcinoma (%)	7 (0.2)	0	7 (0.4)	0.024
Treated acute rejection at discharge	304 (9.0)	159 (10.8)	145 (7.6)	0.002

Continuous variables were summarized as median and interquartile ranges (IQR). Categorical variables are summarized as counts and percentages. Summary statistics excluded records with missing values. BMI = body mass index, DCD = donor after circulatory death, IL-2 = interleukin 2, LDRI = liver donor resk index, MMF = mycophenolate mofetil.

**Table 2:** Characteristics of study population among adult liver transplant recipients with PSC transplanted during February 2002–February 2020 by donor–recipient race.

	Black–Black *n* = 148	Black–White *n* = 371	White–White *n* = 2268	White–Black *n* = 346	*p*-Value
Recipient age Median (IQR)	39 (30, 51)	50 (38, 59)	50 (37, 59)	44 (33, 53)	*p* < 0.001
Recipient Female (%)	66 (44.6)	102 (27.5)	640 (28.2)	155 (44.8)	*P* < 0.001
Allocation MELD (median, IQR)	23 (18, 28)	23 (19, 30)	22 (17, 27)	26 (22, 32)	*p* < 0.001
Deceased donor (%)	123 (83.1)	368 (99.2)	1757 (77.5)	343 (99.1)	<0.001
Living donor (%)	25 (16.9)	3 (0.8)	511 (22.5)	3 (0.9)	<0.001
DCD (%)	1 (0.8)	8 (2.2)	107 (6.1)	11 (3.2)	<0.001
Donor age, median (IQR)	35 (24, 49)	40 (23, 53)	41 (28, 53)	39 (24, 52)	<0.001
LDRI	1.43 (1.25, 1.71)	1.48 (1.26, 1.81)	1.32 (1.08, 1.58)	1.20 (1.05, 1.50)	<0.001
Tacrolimus (%)	144 (97.3)	357 (96.2)	2183 (96.3)	326 (94.2)	*p*=0.226
MMF (%)	128 (86.5)	311 (83.8)	1790 (78.9)	283 (81.8)	*p*=0.022
Treated acute rejection at discharge	19 (13.7)	26 (7.6)	174 (8.8)	29 (9.4)	*p*=0.186

DCD = donor after circulatory death, IQR = interquartile range, MELD = model for end-stage liver disease, MMF = mycophenolate mofetil.

**Table 3:** Results of multivariable analysis for 5-year patient survival adult liver recipients from deceased donors transplanted for PSC during February 2002–September 2018.

	HR [95% CI]	*p* Value
Recipient age (59 vs. 37 years old)	1.56 [1.29, 1.89]	<0.001
Recipient Female	0.88 [0.68, 1.15]	0.351
Donor Female	0.91 [0.71–1.17]	0.471
Donor age (54 vs. 25 years old)	0.98 [0.74, 1.31]	0.906
LDRI 1.63 vs. 1.11	1.03 [0.75, 1.41]	0.864
Transplant era 1 vs. 2	0.88 [0.70, 1.12]	0.307
Treated for acute rejection at discharge	0.94 [0.58, 1.52]	0.796
Donor–Recipient:		
Black–Black vs White–White	1.02 [0.49,2.12]	0.958
Black–White vs White–White	1.27 [0.86, 1.89]	0.233
White–Black vs White–White	1.69 [1.16, 2.45]	0.006
Other Races vs. White–White	1.54 [1.13, 2.11]	0.006

CI = confidence interval, HR = hazard ratio, LDRI = liver donor risk index.

## Data Availability

Data available from organ procurement and transplant network.

## Supplementary Materials

The following supporting information can be downloaded at: https://www.mdpi.com/article/10.3390/jcm14155441/s1, Table S1: STROBE Statement—checklist of items that should be included in reports of observational studies.

## Abbreviations

ACA	affordable care act
D	donor
DCD	donor after circulatory death
MELD	model for end-stage liver disease
OPTN	Organ Procurement and Transplantation Network
PSC	primary sclerosing cholangitis
R	recipient
SRTR	Scientific Registry for Transplant Recipients
UNOS	United Network for Organ Sharing
