# Supplementary material for: Donor–Recipient Race Mismatch Is Associated with Lower Survival After Liver Transplantation for Primary Sclerosing Cholangitis
Source: J Clin Med. 2025 Aug 1;14(15):5441. doi: 10.3390/jcm14155441 (PMC12347405; doi:10.3390/jcm14155441)
Supplement: Supplementary file 1 [file jcm-14-05441-s001.zip › jcm-3746069-supplementary.pdf]

Supplementary Table S1: STROBE Statement—checklist of items that should be included in reports of observational studies

	Item No.	Recommendation	Page No.	Relevant text from manuscript
**Title and abstract**	1	(*a*) Indicate the study's design with a commonly used term in the title or the abstract	3	A retrospective observational study
		(*b*) Provide in the abstract an informative and balanced summary of what was done and what was found	3	
**Introduction**				
Background/rationale	2	Explain the scientific background and rationale for the investigation being reported	4	With the changing demographics in the United States, we sought to report trends and outcomes after liver transplantation for PSC in the United States across two decades
Objectives	3	State specific objectives, including any prespecified hypotheses	4	…based upon prior studies demonstrating differences in outcomes by donor-recipient race after liver transplantation for indications other than PSC, we analyzed outcomes stratified on donor-recipient race after liver transplantation for PSC.
**Methods**				
Study design	4	Present key elements of study design early in the paper	5	We analyzed the OPTN database for adult (≥18-years-old) recipients of deceased donor liver transplants with PSC diagnoses between February 27, 2002 (implementation of MELD) and February 26, 2020…
Setting	5	Describe the setting, locations, and relevant dates, including periods of recruitment, exposure, follow-up, and data collection	5	The primary outcomes were defined as graft failure and death within 5 years after transplant. Follow-up time was measured as the duration from the transplant date to either the occurrence of the primary outcomes or the last known follow-up date.
Participants	6	(*a*) *Cohort study*—Give the eligibility criteria, and the sources and methods of selection of participants. Describe methods of follow-up<br>*Case-control study*—Give the eligibility criteria, and the sources and methods of case ascertainment and control selection. Give the rationale for the choice of cases and controls<br>*Cross-sectional study*—Give the eligibility criteria, and the sources and methods of selection of participants	5	We analyzed the OPTN database for adult (≥18-years-old) recipients of deceased donor liver transplants with PSC diagnoses between February 27, 2002 (implementation of MELD) and February 26, 2020 (prior to COVID pandemic) who were alive with a functioning graft at discharge. Recipients that had a diagnosis of PSC with or without inflammatory bowel

				disease were included. Multiorgan transplants, retransplants and individuals with a prior transplant were excluded.
		(*b*) *Cohort study*—For matched studies, give matching criteria and number of exposed and unexposed<br>*Case-control study*—For matched studies, give matching criteria and the number of controls per case		
Variables	7	Clearly define all outcomes, exposures, predictors, potential confounders, and effect modifiers. Give diagnostic criteria, if applicable	5,6	The primary outcomes were defined as graft failure and death within 5 years after transplant. Follow-up time was measured as the duration from the transplant date to either the occurrence of the primary outcomes or the last known follow-up date. Primary outcomes were assessed for adult deceased donor liver transplants through September 2018, to allow for 5 years of follow-up data plus an additional 3 months due to data reporting lags.
Data sources/ measurement	8*	For each variable of interest, give sources of data and details of methods of assessment (measurement). Describe comparability of assessment methods if there is more than one group	5	OPTN database
Bias	9	Describe any efforts to address potential sources of bias	6	Missing values for covariates were imputed using multiple imputation by chained equations via the aregImpute function from the Hmisc package version 5.1-3 and pooled across 11 imputation datasets for the cohort of deceased donor transplants.
Study size	10	Explain how the study size was arrived at	5	Two transplant eras (2002-2011 and 2012-2020) were used to evaluate changes in characteristics of all PSC transplants over time while still maintaining an adequate number of recipients in each era, including for subgroup analyses

Continued on next page

Quantitative variables	11	Explain how quantitative variables were handled in the analyses. If applicable, describe which groupings were chosen and why	6	Continuous variables were summarized as median and interquartile range (IQR). Distributions of continuous variables were compared by group using a Kruskal-Wallis test. Categorical variables were reported as number and percent
Statistical methods	12	(*a*) Describe all statistical methods, including those used to control for confounding	6	Risk adjustment variables used in the Cox models were identified *a priori* to reduce bias.
		(*b*) Describe any methods used to examine subgroups and interactions	6	Donor and recipient race was classified as White, Black, Hispanic, Asian, and 'Other" in the univariate tabulation. For the outcomes analysis, the Hispanic and Asian categories were combined into 'Other' due to sample size. Additionally, donor-to-recipient race was categorized as White-White, White-Black, and 'Other', with 'Other' encompassing any combinations outside the first four. A donor-recipient mismatch refers to transplant where the race of the donor differs from that of the recipient.
		(*c*) Explain how missing data were addressed	6	Missing values for covariates were imputed using multiple imputation by chained equations via the aregImpute function from the Hmisc package version 5.1-3 and pooled across 11 imputation datasets for the cohort of deceased donor transplants.
		(*d*) *Cohort study*—If applicable, explain how loss to follow-up was addressed<br>*Case-control study*—If applicable, explain how matching of cases and controls was addressed<br>*Cross-sectional study*—If applicable, describe analytical methods taking account of sampling strategy	6	Follow-up time was measured as the duration from the transplant date to either the occurrence of the primary outcomes or the last known follow-up date.
		(*e*) Describe any sensitivity analyses		N/A
**Results**				
Participants	13*	(a) Report numbers of individuals at each stage of study—eg numbers potentially eligible, examined for eligibility, confirmed eligible, included in the study, completing follow-up, and analysed	7-8, Table 1	--<br>--<br>--<br>--<br>--<br>--
		(b) Give reasons for non-participation at each stage		

		(c) Consider use of a flow diagram	
Descriptive data	14*	(a) Give characteristics of study participants (eg demographic, clinical, social) and information on exposures and potential confounders	Table 1
		(b) Indicate number of participants with missing data for each variable of interest	P 6<10.9%
		(c) *Cohort study*—Summarise follow-up time (eg, average and total amount)	All recipients followed for 5 years
Outcome data	15*	*Cohort study*—Report numbers of outcome events or summary measures over time	P7
		Case-control study—Report numbers in each exposure category, or summary measures of exposure	
		Cross-sectional study—Report numbers of outcome events or summary measures	
Main results	16	(*a*) Give unadjusted estimates and, if applicable, confounder-adjusted estimates and their precision (eg, 95% confidence interval). Make clear which confounders were adjusted for and why they were included	P 7-8, Figures 1,2, Table 2,
		(*b*) Report category boundaries when continuous variables were categorized	
		(*c*) If relevant, consider translating estimates of relative risk into absolute risk for a meaningful time period	

Continued on next page

Other analyses	17	Report other analyses done—eg analyses of subgroups and interactions, and sensitivity analyses	P8	
**Discussion**				
Key results	18	Summarise key results with reference to study objectives	P9-10	
Limitations	19	Discuss limitations of the study, taking into account sources of potential bias or imprecision. Discuss both direction and magnitude of any potential bias	P10 2nd full paragraph	
Interpretation	20	Give a cautious overall interpretation of results considering objectives, limitations, multiplicity of analyses, results from similar studies, and other relevant evidence	P11, ABSTRACT findings warrant further study	
Generalisability	21	Discuss the generalisability (external validity) of the study results	P 10	We did not validate our findings in an independent cohort of recipients and our findings of donor-recipient race mismatch on patient survival warrants validation prior to reaching definitive conclusions
**Other information**				
Funding	22	Give the source of funding and the role of the funders for the present study and, if applicable, for the original study on which the present article is based	Study supported by unrestricted funds from the Anne and Epes Robinson Liver Research Fund	

*Give information separately for cases and controls in case-control studies and, if applicable, for exposed and unexposed groups in cohort and cross-sectional studies.

Note: An Explanation and Elaboration article discusses each checklist item and gives methodological background and published examples of transparent reporting. The STROBE checklist is best used in conjunction with this article (freely available on the Web sites of PLoS Medicine at http://www.plosmedicine.org/, Annals of Internal Medicine at http://www.annals.org/, and Epidemiology at http://www.epidem.com/). Information on the STROBE Initiative is available at www.strobe-statement.org.
